# Method for the simplistic modelling of the acoustic footprint of the vessels in the shallow marine area^☆^

**DOI:** 10.1016/j.mex.2018.08.011

**Published:** 2018-08-28

**Authors:** Donatas Bagočius, Aleksas Narščius, Olga Anne

**Affiliations:** a Marine Research Institute, Klaipėda University, H. Manto 84a, Klaipėda, Lithuania; b Faculty of Marine Technology and Natural Sciences, Department of Natural Sciences, Klaipėda University, H. Manto str. 84, 92294 Klaipėdaa, Lithuania

**Keywords:** Simplistic modelling of the vessels acoustic footprint in the shallow marine area, Underwater noise, Shipping, Modelling

## Abstract

The definitions of the 11th descriptor of the EU Marine Strategy Framework Directive (MSFD) “Underwater noise and other forms of energy” outlines the standards for the continuous noise evaluation and monitoring in the European seas. Long lasting fluctuations of the continuous underwater noise at the shallow marine areas in the low frequency bands (<1 kHz) are mostly associated with the shipping noise, where these fluctuations are sensitive to changes in the spatial distribution of human activities, or changes of environmental and climatic variables. Underwater noise modelling is usually considered as a supplement to noise measurements, where models increases the utility of the measurement results. Noise mapping is considered as a form of spatial modelling, providing a convenient and accessible way to visualise models. Therefore, underwater noise models and maps can be used in management and evaluation of environmental state. There are number of freely available widely used noise source and sound propagation models. Still the simplistic logarithmic rules purposed for the sound propagation loss computations do not account for the number of factors in the marine environment, i.e. sediment type, water depth or frequency. On the other hand the sophisticated physical models purposed for the description of the footprint of noise sources such as ships are complex and their programing requires very specific knowledge. In this paper the details of the method purposed for modelling of the ship noise footprint in shallow seas is presented. Proposed method allows to compute:

• depth dependent ship sound transmission losses in 1 Hz frequency bands;
• sound propagation losses during different seasons (summer/winter);
• acoustic footprint accounting for vessel noise directivity.

## Graphical abstract

**Figure:**
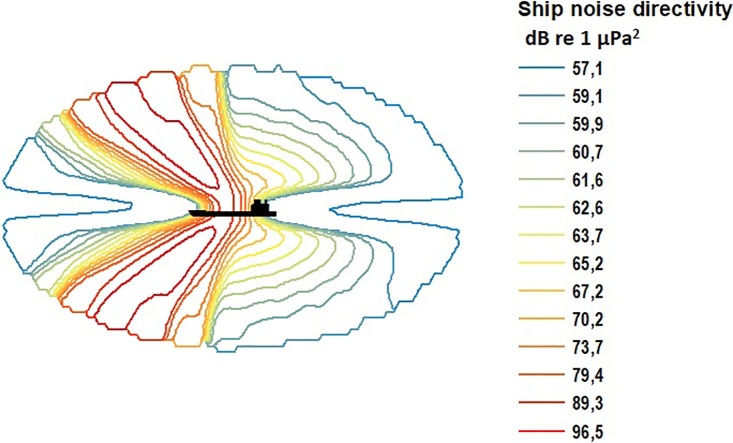

**Specifications Table**

**Table:** 

Subject area	• *Environmental Science*
More specific subject area	*Underwater acoustics*
Method name	*Simplistic modelling of the vessels acoustic footprint in the shallow marine area*

## Method details

### The research area and the hydro-acoustic properties

**Fig. 1:**
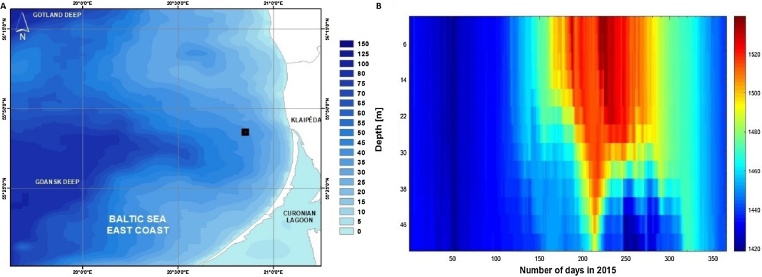
A – Bathymetry of the Lithuanian Baltic Sea area (black square marks the location of the data acquisition from the hydrodynamic model); B – daily sound velocity profile for the period of 2015, acquired from the hydrodinamic model, colour bar marks SVP in m/s.

Lithuanian marine area is located at the Eastern part of the Baltic Sea coast in the Gotland basin [1]. Lithuanian marine areas borders with the Russian Federation in the southern part, in the North with Latvia, and in the West with Sweden. At the centre of the Lithuanian EEZ the Nemunas Palaeo opens to the inside of the Gdansk Peninsula [2]. A Klaipeda–Ventspils Plateau gradually slopes through the Gdansk sill to the West forming the deeper areas having the depths of >60 m reaching ∼125 m at the West [3], where the downslope from the East to West has no any steep ridges or cliffs forming favourable sound propagation paths. The bathymetry of the Lithuanian EEZ depicted in the Fig. 1A.

For description of sound propagation conditions and determination of winter ducting period at Lithuanian EEZ the SVP’s were computed using TS data for the year of 2015. The TS data were acquired from the EU Marine Environment Monitoring Service database (Baltic Sea Physics Reanalysis From SMHI 1989–2015 HIROMB model, [4]), acquired at the location 55°43′8.32″N; 20°36′40.31″E (see Fig. 1A), using the equation:

(1) $$C_{w}\left(z\right)=1449.2+4.6T-0.055T^{2}+0.00029T^{3}+(1.34-0.01T)(S-35)+0.016H$$

Where *Cw(z)* – depth dependant sound velocity (m/s), *T* – temperature, *S* – salinity, *H* – water depth [5].

SVP data analysis (Fig. 1B) revealed positive sound velocity profiles in the winter season (the period of 2015 January–February), were the sound propagation conditions with the winter surface ducting are formed. In this period the change of sound velocity gradients counted as between the values of 0.03–0.125 s^−1^ (the value of sound change by 1 m/s per 1 m depth see [6]). During the rest of the year (spring–summer–autumn seasons), the sound velocity profiles assumed to be an ISO and negatively inclined, where the sound propagation taking place in the “mode stripping” region, with extensive surface-bottom interaction of sound waves [7].

The dominating bottom sediments in the coastal Lithuanian marine area are the sandy and aleuritic substrate. Central part of the EEZ is covered mainly by coarse aleurite and western deepest part of EEZ is covered mostly with pelitic and pelitic-aleuritic mud. Still, at the Lithuanian EEZ and coastal areas the sandy bottom substrates dominate [8]. For the computations of sound transmission losses the bottom sediments assumed to be sandy and silty substrate throughout the Lithuanian marine area.

### Ship source levels

**Fig. 2:**
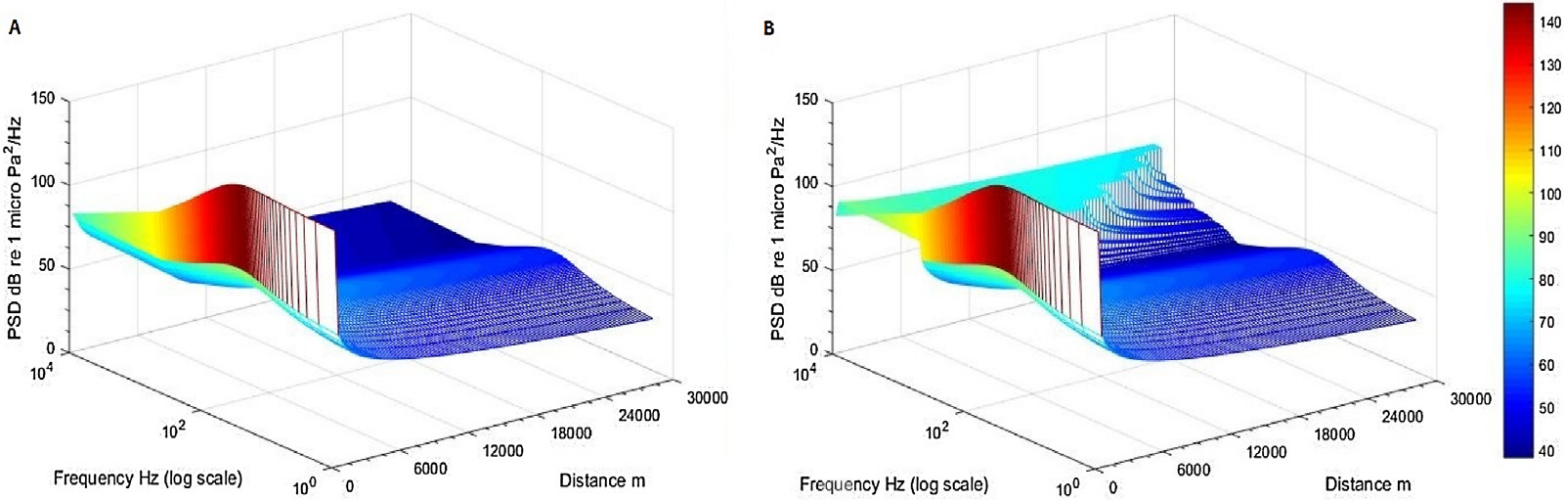
Modelled sound propagation in different seasons with custom shoaling bathymetry (elevation angle a = 0.08°, A – summer season, B – winter season (with appearing winter ducting above 600 Hz – light blue colour), source level 160.35 dB re 1μPa^2^ m^2^. Colour bar marks power spectral density levels in dB re 1 μPa^2^/Hz.

To compute shipping noise levels, three entities are in generally required; density of shipping, the source level of the ships, and the transmission loss [9]. These computations inevitably will vary due to the different locations and different seasons [10]. Source levels of vessels are computed in 1 Hz frequency bands (Fig. 2 Y & Z axes), using the Research Ambient Noise Directionality Model 3.1 algorithm (for description see [11]). The ship source levels assumed to be dipole, “surface modified” radiated noise levels [7].

### Sound propagation modelling

For sound transmission loss modelling, the algorithm by Ainslie et al. [7] was used where the three propagation scenarios are modelled.

Three propagation scenarios:

**Spherical** propagation loss, equation:

(2) $${TL}_{SP}=40{Log}_{10}\left(\frac{r}{r_{0}}\right)-10{Log}_{10}[4({\frac{kzD}{r_{0}})}^{2}]$$

**Cylindrical** propagation loss equation:

(3) $${TL}_{cyl}=10{Log}_{10}\left(\frac{r}{r_{0}}\right)+10{Log}_{10}[\frac{3H}{\left(k^{2}z^{2\;}\beta_{0}^{3}r_{0}\right)}]$$

Propagation loss in the **Mode stripping** area:

(4) $$TL_{Mds}=25Log_{10}\left(\frac{r}{r_{0}}\;\right)+10Log_{10}\left[4{\left(kz\right)}^{-2}{\left(\frac{{\text{η}}^{3}r_{0}}{\pi H}\right)}^{1/2}\right]$$

Where the *r* is a distance, *r*_0_ – reference distance (1 m), *k* – wavenumber, *z* – source depth, *D* – receiver depth (assumed to be H/2 at any location), *H* – water depth, *β* – critical angle, *ƞ* – constant for sandy and silty substrate (0.3 Np/rad).

The cylindrical and mode stripping sound transmission loss computations were implemented at 1 Hz frequency bands across the frequency range of 10 Hz – 10 kHz including depth dependence “H” at each range step.

Sound transmission loss equations divided in to two values of A + B i.e. A = 25log_10_(x); B = 10log_10_(y), where TL = A + B = 25log_10_(x) + 10log_10_(y). Any case of sound propagation losses (either cylindrical or mode stripping) are summed at discrete ranges (range steps 300 m). Then computations implemented as a summation of the values of A and B, which are denoted as TL_A_ and TL_B_:

(5) $${TL}_{r}={TL}_{Ar}+\;{TL}_{Br}$$

The TL_Ar_ equals to:

(6) $${TL}_{Ar}=\;\sum_{i=0}^{n}a_{i}=\;10{Log}_{10}(10^{\frac{a_{1}}{10}}+10^{\frac{a_{2}}{10}\;}\ldots \ldots +10^{\frac{a_{n}}{10}})$$

The TL_Br_ equals to:

(7) $${TL}_{Br}=\;\sum_{i=0}^{n}b_{i}=\;10{Log}_{10}(10^{\frac{b_{1}}{10}}+10^{\frac{b_{2}}{10}\;}\ldots \ldots +10^{\frac{b_{n}}{10}})$$

Where values *a_i_* and *b_i_* are the sound transmission loss values at each consecutive discrete range step.

Transmission loss computations in the different seasons (winter/summer) in the range of 0–300 m consisted of summation of losses in different sound propagation regions (spherical + cylindrical/mode stripping) and at the ranges above 300 m summing the transmission losses of equal propagation (using the equation No. (5)):

In the summer season (January–February):

(8) $${TL}_{summer}={TL}_{Sp}+\;{TL}_{Mds}$$

In the winter season (rest of the year):

(9) $${TL}_{winter}={TL}_{Sp}+\;{TL}_{Cyl}$$

The transition range between spherical sound transmission losses and the losses in the mode stripping region or cylindrical propagation regions were equated using the equation:

(10) $$r_{t}=\frac{H}{2\text{tan}\beta {}^{\circ}}$$

Where *r_t_* – transition range between spherical propagation and propagation of sound waves in the entire shallow sound channel, ensonified in a cylindrical mode, *H* – water depth, *β°* – the critical grazing angle of sound propagation [12]. Critical grazing angle defined by:

(11) $$\beta {}^{\circ}=\text{arccos}\left(\frac{v_{1}}{v_{2}}\right)\;$$

Where *v*_1_ – sound speed in water, *v*_2_ – sound speed in sediments [13]. Sound transmission losses in winter season while computed where filtered using pass-band filter, where winter ducting cut- off frequencies were defined using the equation:

(12) $$f_{o}=\frac{1500}{0.008\;H^{\frac{3}{2}}}$$

Where *f*_o_ – cut-off frequency in Hz below which sound cannot propagate in the duct [13].

Consequentially sound propagation in the spectra of the frequencies above the cut-off frequency were computed using winter transmission loss equation ***TL*_winter_** and the remaining frequencies of spectra using the ***TL*_summer_** transmission loss equation. An example of two modelled different seasonal propagation scenarios presented in the Fig. 2. In the Fig. 2B is clearly visible the sound propagation trapped in the winter duct above the 600 Hz extending to the great distance.

As well, model was programmed to account for sound leakage from the duct [13] at the shoaling bathymetry, where transmission loss values are conserved as the highest loss values, while reaching the lowest depths at the sound wave paths.

In the areas with the shoaling bottom, model was programed to filter noise spectra with a pass band filter applying the cut-off frequency equation:

(13) $$fc=\;\frac{v_{1}/4H}{\sqrt{1-\;v_{1}^{2}/v_{2}^{2}}}$$

Where *fc* is the cut-off frequency in the shallow water [14].

**Fig. 3:**
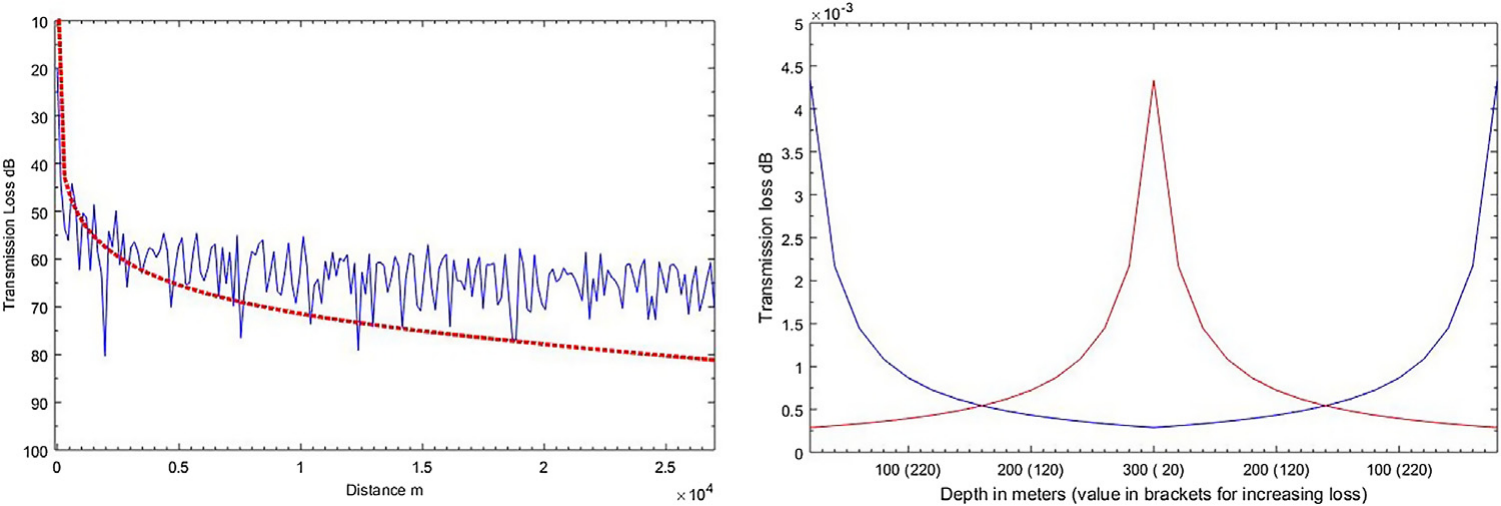
Panel A - comparison of summer transmission losses with the results modelled using KRAKEN (NM) model at 100 Hz frequency, fixed receiver depth 20 m, with custom shoaling bathymetry, elevation angle α ≈ 0.08°, Panel B – Depth dependence of the sound transmission loss equation in the mode stripping region (at 100 Hz frequency, sandy bottom), blue line in the figure: propagation loss depth dependence with the depth varying from 20 to 300 m (decreasing loss); red line propagation loss depth dependence with the depth varying from 300 to 20 m depth (increasing loss).

Modelled summer sound transmission losses were compared with the Normal Mode sound propagation loss model KRAKEN [15] and sound transmission loss model was tested for depth dependence. The comparison and test results are presented in the Fig. 3.

Computed summer transmission losses were compared with the transmission losses computed using the Normal Mode model in 100 Hz frequency band at the different receiver depths above and below the thermocline (summer sound speed profile), computed with the shoaling bathymetry. The comparison presented in the Table 1.

**Table 1:** Comparison of deviation of computed transmission losses from the results modelled using NM model at different receiver depths (above/below thermocline).

Receiver depth (above/below thermocline)	Distance to the noise source			
	300 m	1200 m	4800 m	19200 m
15 m	−10.42 dB	+1.97 dB	+1.43 dB	+12.70 dB
40 m	−5.21 dB	−2.28 dB	+5.27 dB	+12.72 dB
Difference	5.21 dB	4.25 dB	3.84 dB	0.02 dB

Greater deviations of computed transmission losses from the losses computed using NM model were observable at the greater distances at both scenarios (>15 km).

### Computations of the surface acoustic footprint

For the spatial noise distribution computations around the vessel, the transmission losses along uncoupled azimuthal planes of length Δr = 20 nm (distance chosen for experimental purposes), allowing the construction of an acoustic image around the noise sources was used. The vertical radials of sound propagation during computations are separated by the angle φ, yielding 32 bearings at every 11.25° (360°/φ) at each noise source position (see [13]).

Noise propagation directivity was computed as well, using the coefficients of sound propagation pattern of merchant vessel example (sailing at 8 kn speed) in the frequency band of 4 kHz, expressed as the sound pressure ratio between the highest-pressure levels radiated at the ships abeam and the rest of the noise propagation directions. Sound pressure levels around the noise source then were multiplied by obtained coefficients. Radiated acoustic energy is computed as greatest at abeam direction and noise radiated astern is weakened by bubbly wake and in forward direction reduced by ship’s hull [16]. The directivity coefficients presented in the Table 2.

**Table 2:** Directivity coefficients derived from data by [16].

Azimuthal mid angle, deg	P^2^/Hz (Pascals^2^)	Ratio of pressures in dB re 1 μPa^2^
0	0	0,000000000000
7,5	9	0,599363585154
30	64	0,624694030188
60	100	0,630456832882
90	144	0,635165385526
120	256	1,000000000000
150	144	0,635165385526
172	16	0,606793130002
180	0	0,000000000000

**Fig. 4:**
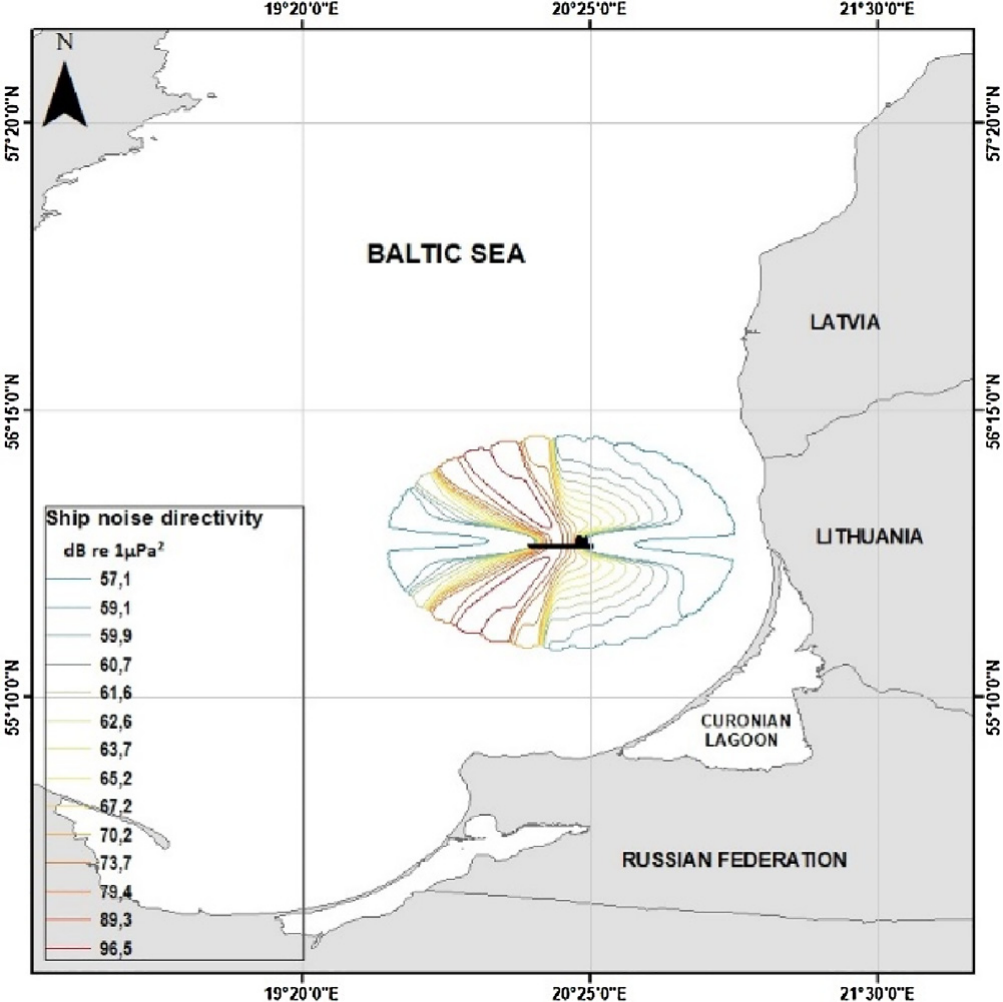
Computed depth and frequency dependent directional broadband ship noise footprint (isolines marks sound pressure levels at 32 azimuthal bearings).

The sound pattern directivity of the merchant vessel radiating the broadband surface sound pressure level equal to 171.3 dB re 1 μPa^2^ m^2^ at the distance of 48 km from the land sailing at the 9.2 knot speed, plotted as the geographical map in the Fig. 4.

Acquired noise data of ships then can be integrated in to the spatial grid to build acoustic footprint in the area of interest and the soundscape surface maps can be derived using the interpolation techniques [17].
